# The Effectiveness of Serious Games on Cognitive Processing Speed Among Older Adults With Cognitive Impairment: Systematic Review and Meta-analysis

**DOI:** 10.2196/36754

**Published:** 2022-09-09

**Authors:** Alaa Abd-alrazaq, Arfan Ahmed, Haitham Alali, Ahmad Mohammad Aldardour, Mowafa Househ

**Affiliations:** 1 AI Center for Precision Health Weill Cornell Medicine-Qatar Doha Qatar; 2 Health Management Department, Faculty of Medical and Health Sciences Liwa College of Technology Abu Dhabi United Arab Emirates; 3 Physical Therapy Department Rumailah Hospital Hamad Medical Corporation Doha Qatar; 4 Division of Information and Computing Technology, College of Science and Engineering Hamad Bin Khalifa University Qatar Foundation Doha Qatar

**Keywords:** serious games, cognitive training, exergames, processing speed, mild cognitive impairment, Alzheimer disease, dementia, systematic reviews, meta-analysis, mobile phone

## Abstract

**Background:**

Human cognitive processing speed is known to decline with age. Human cognitive processing speed refers to the time that an individual takes from receiving a stimulus to reacting to it. Serious games, which are video games used for training and educational purposes, have the potential to improve processing speed. Numerous systematic reviews have summarized the evidence regarding the effectiveness of serious games in improving processing speed, but they are undermined by some limitations.

**Objective:**

This study aimed to examine the effectiveness of serious games on the cognitive processing speed of an older adult population living with cognitive impairment.

**Methods:**

A systematic review of randomized controlled trials (RCTs) was conducted. Two search sources were used in this review: 8 electronic databases and backward and forward reference list checking. A total of 2 reviewers independently checked the eligibility of the studies, extracted data from the included studies, and appraised the risk of bias and quality of the evidence. Evidence from the included studies was synthesized using a narrative and statistical approach (ie, meta-analysis), as appropriate.

**Results:**

Of the 548 publications identified, 16 (2.9%) RCTs eventually met all eligibility criteria. Very-low-quality evidence from 50% (8/16) and 38% (6/16) of the RCTs showed no statistically significant effect of serious games on processing speed compared with no or passive intervention groups (*P*=.77) and conventional exercises (*P*=.58), respectively. A subgroup analysis showed that both types of serious games (cognitive training games: *P*=.26; exergames: *P*=.88) were as effective as conventional exercises in improving processing speed.

**Conclusions:**

There is no superiority of serious games over no or passive interventions and conventional exercises in improving processing speed among older adults with cognitive impairment. However, our findings remain inconclusive because of the low quality of the evidence, the small sample size in most of the included studies, and the paucity of studies included in the meta-analyses. Therefore, until more robust evidence is published, serious games should be offered or used as an adjunct to existing interventions. Further trials should be undertaken to investigate the effect of serious games that specifically target processing speed rather than cognitive abilities in general.

**Trial Registration:**

PROSPERO CRD42022301667; https://www.crd.york.ac.uk/prospero/display_record.php?RecordID=301667

## Introduction

### Background

By 2050, the older adult population is expected to double on a global scale [1] followed by a growing demand for high-quality support among this population. There is a variety of disabilities related to aging. However, a considerable percentage of people aged >65 years are affected by cognitive impairments. According to the Alzheimer’s Association, approximately 15% to 20% of people aged >65 years are affected by mild cognitive impairment (MCI) [2]. It is a progressive, terminal brain disorder for which the cure and cause are unknown [3]. Worldwide, the number of Alzheimer disease (AD) cases is rising; estimates indicate that 44 million people currently have AD or dementia. As the Baby Boomer generation passes the age of 65 years, the number of people experiencing AD is expected to reach 76 million by 2030 [4,5]. The consequences of these cognitive disorders include poor judgment, decreased activity, and memory loss. They are difficult to manage; thus, it would be highly beneficial to develop proper and effective interventions for the older adult population.

These disorders imply a decline in various cognitive functions such as memory [6,7], executive function [8,9], attention [10], and processing speed [11]. Processing speed refers to the time that an individual takes from receiving a stimulus to reacting to it. Slowed processing speed is a typical characteristic of cognitive aging. It implies a slowing rate at which individuals perform decision-making as well as motor and perceptual tasks. Moreover, processing speed represents one of the critical predictors of performance related to cognitive tasks in older adults; it is the basis of a primary hypothesis for age-related cognitive decline [11,12]. Accordingly, aging research primarily aims to design methods for improving and maintaining cognitive functions in older adults.

Serious games are one of the interventions that have been used to improve and maintain processing speed in older adults. Serious games represent a type of video game used for training and educational purposes in different contexts [13,14]. Serious games have shown promising results in improving cognitive functions such as global cognition [15-18], processing speed [16-20], memory [16-23], executive function [16-22,24], and attention [16,17,19,20,22]. The most common types of serious games used for improving cognitive abilities are exergames (video games that require physical exercise as part of playing) and cognitive training games (video games that include cognitively stimulating activities designed to maintain or promote the users’ cognitive abilities).

### Research Gap and Aim

Several studies have explored the therapeutic impact of serious games on processing speed. However, the evidence from these studies is still fragmented. Aggregating the evidence through systematic reviews is important to draw conclusions about the effectiveness of serious games in improving processing speed. A total of 5 systematic reviews have pooled findings from these studies, but they are undermined by some limitations [16-20]. Specifically, these reviews (1) focused on older adults without cognitive impairment [16,18-20], (2) included quasi-experiments or pilot randomized controlled trials (RCTs) [17-19], (3) did not assess the quality of the meta-analyzed evidence [17,18,20], (4) only focused on a specific type of serious game such as cognitive training games [16,17,20] and exergames [18], or (5) did not compare the effect of serious games with a specific comparator (eg, no intervention, conventional exercises, or conventional cognitive activities) [16-18,20]. Accordingly, the aim of this review was to examine the effectiveness of serious games on the cognitive processing speed of an older adult population living with cognitive impairment. To address the aforementioned limitations, we (1) focused on older adults with cognitive impairment; (2) included only RCTs; (3) appraised the quality of the meta-analyzed evidence using the Grading of Recommendations Assessment, Development, and Evaluation (GRADE) approach; (4) included all types of serious games; and (5) compared the effect of serious games with that of a specific comparator. In this review, effectiveness refers to the degree to which serious games are successful in improving cognitive processing speed.

## Methods

To conduct this systematic review, we followed the PRISMA (Preferred Reporting Items for Systematic Reviews and Meta-Analyses) guidelines (Multimedia Appendix 1) [25]. The protocol for this review is registered at PROSPERO (ID: CRD42022301667).

### Search Strategy

#### Search Sources

The first author searched the following databases on November 10, 2021: MEDLINE (via Ovid), PsycINFO (via Ovid), Embase (via Ovid), CINAHL (via EBSCO), IEEE Xplore, ACM Digital Library, Scopus, and Google Scholar. Only the first 10 pages (ie, 100 hits) were considered as the databases return a large number of studies automatically ordered according to relevance [26]. Finally, backward reference list checking (ie, screening the reference lists of the included studies and relevant reviews) and forward reference list checking (ie, screening the studies that cited the included studies) were conducted.

#### Search Terms

The search query was developed in consultation with 2 experts in digital mental health. It included terms related to the target population (eg, cognitive disorder), target intervention (eg, serious games), and targeted study design (eg, RCTs). Multimedia Appendix 2 summarizes the search query that was used for each of the 8 databases.

### Study Eligibility Criteria

Only RCTs that evaluated the effectiveness of serious games in improving processing speed among older adults with cognitive impairment were included in this study. To be more precise, we considered studies that included serious games available on any digital platform, such as PCs, video game consoles (eg, Xbox and PlayStation), mobile phones, tablets, handheld devices, Nintendo, or any other type of digital device. The game had to be the major component of the intervention and used solely for therapeutic purposes. Studies involving serious games in combination with other interventions were included if the control group underwent the same adjacent intervention. Games that were not based on digital technology (eg, paper-and-pencil games and board games) or that were used for monitoring, screening, diagnosis, or research were excluded.

The study population was older adults (aged ≥60 years) with any type of cognitive impairment or disorder as confirmed by checking the inclusion criteria or baseline scores against defined diagnostic criteria (eg, Mini-Mental State Examination). Older adults without cognitive impairment, health care providers, and caregivers were beyond the scope of this review. No restrictions were applied regarding gender or ethnicity.

The outcome of interest in this review was cognitive processing speed. No restrictions were applied regarding the outcome measures. This review did not consider studies that focused only on cost-effectiveness, acceptance, feasibility, satisfaction, or cognitive abilities other than processing speed. The focus of this review was on postintervention data (ie, outcome data collected just after the intervention) rather than follow-up data (ie, outcome data collected a period after the intervention).

All types of RCTs (ie, parallel, cluster, crossover, and factorial) were considered in this review, whereas pilot RCTs, quasi-experiments, observational studies, and reviews were excluded. We included journal articles, conference proceedings, and dissertations, whereas abstracts, conference posters, commentaries, proposals, and editorials were excluded. This review was restricted to only studies written in the English language and published since 2010. We did not apply restrictions on the country of publication, comparator, or study settings.

### Study Selection

We identified relevant studies by using the following process. First, all the retrieved studies were imported into EndNote (Clarivate Analytics) to find duplicate publications and remove them. Second, the titles and abstracts of all the retrieved studies were checked by 2 reviewers working independently (the first and second authors). Finally, both reviewers independently read the complete texts of the studies included in the previous step. Any disagreements were resolved via discussion. Steps 2 and 3 had an interrater agreement (Cohen κ) of 0.88 and 0.96, respectively.

### Data Extraction

Before extracting the data, we pilot-tested the data extraction form with 2 of the included studies. Microsoft Excel was used by 2 reviewers (the first and second authors) to independently extract data from the included studies. Any disputes in the extracted data between the reviewers were resolved through discussion. The first and corresponding authors of the included studies were contacted to retrieve outcome data (eg, mean, SD, and sample size) if they were missing from the published articles. The data extraction form is provided in Multimedia Appendix 3.

### Risk of Bias Appraisal

Two reviewers (the first and second authors) independently examined the risk of bias in the included studies using the Risk of Bias 2 tool [27]. This tool evaluates the risk of bias in 5 areas of RCTs: randomization process, deviations from intended interventions, missing outcome data, measurement of the outcome, and selection of the results [27]. The reviewers held discussions to resolve any disagreements, and the interrater agreement was 0.85.

### Data Synthesis

Narrative and statistical methods were used to summarize the collected data. In our narrative synthesis, we used text and tables to describe the characteristics of the included studies (study metadata, population, interventions, comparisons, and outcome measures). The results of the experiments were compiled and classified according to the comparator: no or passive interventions, conventional exercises, and other serious games. When 2 or more studies from the same comparator submitted sufficient data (ie, mean, SD, and number of participants in each intervention group), meta-analyses were performed using Review Manager (RevMan 5.4; The Cochrane Collaboration). As the type of data for the outcome of interest (processing speed) was continuous and the instruments used to evaluate the outcome varied across the included trials, the standardized mean difference (SMD; Cohen *d*) was used to estimate the overall effect of each study. We also chose the random effects model for the analysis because of the high clinical heterogeneity among the meta-analyzed trials in terms of serious game characteristics (eg, type, duration, frequency, and period), population characteristics (eg, sample size, mean age, and health condition), and outcome measures (ie, tools and follow-up period).

To assess the degree and statistical significance of heterogeneity in the meta-analyzed studies, we calculated *I*^2^ and a chi-square *P* value, respectively. A chi-square *P* value of ≤.05 indicated heterogeneous meta-analyzed studies [28]. When *I*^2^ ranged from 0% to 40%, 30% to 60%, 50% to 90%, and 75% to 100%, the degree of heterogeneity was considered as insignificant, moderate, substantial, or considerable, respectively [28].

We used the GRADE approach [29] to appraise the overall quality of the evidence resulting from the meta-analyses. The GRADE approach examines the quality of evidence based on 5 domains: risk of bias, inconsistency (ie, heterogeneity), indirectness, imprecision, and publication bias [29]. The quality of the meta-analyzed evidence was independently assessed by 2 reviewers (the first and second authors). Any differences between the reviewers were resolved via discussion, and the interrater agreement of the reviewers was 0.94 [30].

## Results

### Search Results

**Figure 1:**
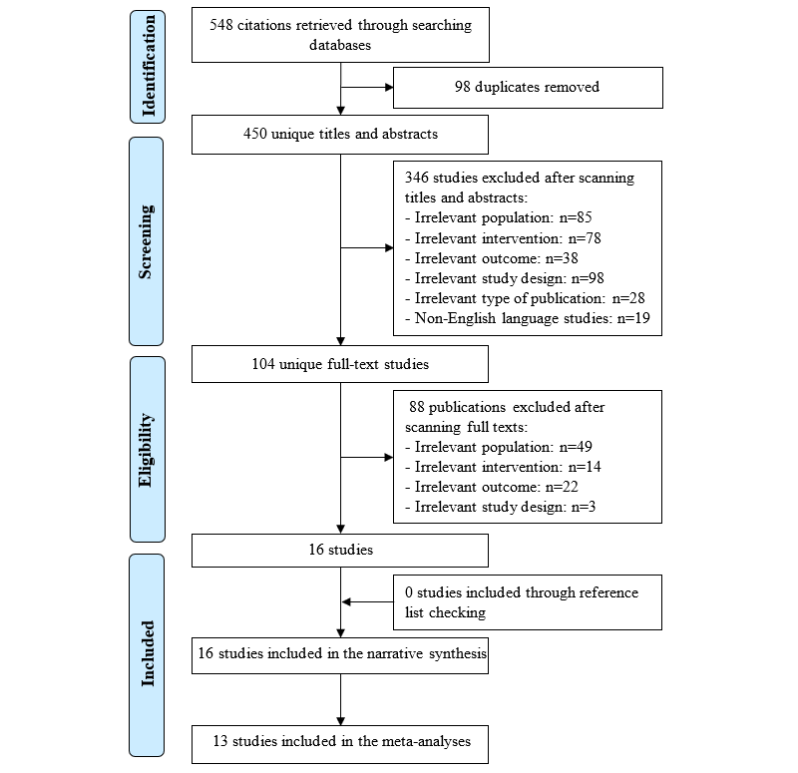
Flowchart of the study selection process.

As shown in Figure 1, a total of 548 records were found after searching 8 electronic databases. Using the EndNote software, of the 548 records, 98 (17.9%) duplicates were removed. Checking the titles and abstracts of the remaining records resulted in the exclusion of 63.1% (346/548) for several reasons shown in Figure 1. Reading the full text of the remaining 104 publications resulted in the exclusion of 88 (84.6%) studies (Figure 1). This review included 16 RCTs in total [31-46]. Of these 16 RCTs, 13 (81%) were included in the meta-analyses [31-43].

### Characteristics of the Included Studies

The included studies were published between 2012 and 2021 and came from 11 different countries (Table 1). Except for a book chapter, all the included papers (15/16, 94%) were peer-reviewed articles. Parallel RCTs were the most common type of trial used in the included studies (14/16, 88%). The sample sizes of the included studies ranged from 20 to 195, with an average of 79.9. The average age of the participants was reported in 94% (15/16) of the studies and varied between 66 and 82.9 years, with an average of 75 years. Male participants in 94% (15/16) of the studies ranged from 23.5% to 71%, with an average of 47.5%. The mean Mini-Mental State Examination score of the participants was reported in 88% (14/16) of the studies and ranged from 18.6 to 28.1, with an average of 24.4. The most common disorder among the participants in the included studies was MCI (11/16, 69%). Participants were drawn from clinical (10/16, 62%), community (4/16, 25%), and clinical and community (2/16, 12%) settings.

**Table 1:** Characteristics of the studies and populations (N=16).

Study, year	Country	Publication type	RCT^a^ type	Sample size	Age, mean (SD)	Male participants, n (%)	MMSE^b^ score	Health condition	Setting
Finn and McDonald [31], 2015	Australia	Journal article	Parallel	31	75.6	22 (71)	28.1	MCI^c^	Clinical
Robert et al [32], 2020	France	Journal article	Parallel	46	79.4	22 (47.8)	21.4	Neurocognitive disorders	Clinical
Savulich et al [33], 2017	United Kingdom	Journal article	Parallel	42	76.1	25 (59.5)	26.7	MCI	Clinical
Valdes et al [34], 2012	United States	Journal article	Parallel	195	77.7	65 (33.3)	NR^d^	MCI	Clinical
Yang and Kwak [35], 2017	South Korea	Journal article	Parallel	20	71	14 (70)	23.1	AD^e^	Clinical
Thapa et al [36], 2020	South Korea	Journal article	Parallel	68	72.7	16 (23.5)	26.2	MCI	Clinical
Tarnanas et al [37], 2014	Greece	Book chapter	Parallel	114	70.3	44 (39)	26.4	MCI	Clinical
Fiatarone Singh et al [38], 2014	Australia	Journal article	Factorial	100	70.1	32 (32)	27	MCI	Community
Amjad et al [39], 2019	Pakistan	Journal article	Parallel	44	NR	NR	24	MCI	Clinical
Wiloth et al [40], 2017	Germany	Journal article	Parallel	99	82.9	28 (28.3)	22	Dementia	Clinical and community
Van Santen et al [41], 2020	Netherlands	Journal article	Cluster	112	79	60 (53.5)	18.6	Dementia	Clinical
Karssemeijer et al [42], 2019	Netherlands	Journal article	Parallel	115	79.9	62 (53.9)	22.4	Dementia	Clinical and community
Liao et al [43], 2021	Taiwan	Journal article	Parallel	61	81.5	20 (32.6)	22.9	MCI	Community
Flak et al [44], 2019	Norway	Journal article	Parallel	85	66	57 (66.7)	NR	MCI	Clinical
Hyer et al [45], 2016	United States	Journal article	Parallel	68	75.2	32 (47.1)	26	MCI	Community
Park and Park [46], 2017	South Korea	Journal article	Parallel	78	67.3	42 (53.8)	26.5	MCI	Community

^a^RCT: randomized controlled trial.

^b^MMSE: Mini-Mental State Examination.

^c^MCI: mild cognitive impairment.

^d^NR: not reported.

^e^AD: Alzheimer disease.

We identified 16 distinct serious games used in the studies (Table 2). More than one game was used in 6% (1/16) of the studies. The serious games in the trials were divided into 2 categories based on the treatment modality they provided: cognitive training games (12/16, 75%) and exergames (4/16, 25%). In 88% (14/16) of the studies, the games were designed with a “serious” objective from the start (designed serious games). By contrast, games in the remaining 12% (2/16) of the studies were not planned as serious games from the start but were instead used for a serious purpose (purpose-shifted games). Serious games were played under the supervision of health care providers or caregivers in most studies (11/16, 69%). The game duration in the included studies ranged from 25 to 100 minutes. The frequency of playing the games ranged from 2 to 7 times per week, but it was <4 times per week in approximately two-thirds of the studies (11/16, 69%). The duration of the interventions ranged from 4 to 25 weeks, but it was <13 weeks in three-fourths of the studies (13/16, 81%).

**Table 2:** Characteristics of the interventions (N=16).

Study	Serious game name	Serious game type	Serious game genre	Platform	Supervision	Duration (minutes)	Frequency (times per week)	Period (weeks)
Finn and McDonald [31]	E-Prime	Cognitive training game	Designed	PC	Supervised	NR^a^	2	4
Robert et al [32]	MeMo	Cognitive training game	Designed	PC and tablet	Unsupervised	30	4	12
Savulich et al [33]	Game Show	Cognitive training game	Designed	Tablet	Supervised	60	2	4
Valdes et al [34]	SOPT	Cognitive training game	Designed	PC	Supervised	60	2	5
Yang and Kwak [35]	Brain-Care	Cognitive training game	Designed	PC	Unsupervised	60	2	12
Thapa et al [36]	Juice Making, Crow Shooting, Love House, and Fireworks	Cognitive training game	Designed	VR^b^ headset and hand controllers	Supervised	100	3	8
Tarnanas et al [37]	Virtual Reality Museum	Cognitive training game	Designed	VR headset	Supervised	90	2	21
Fiatarone Singh et al [38]	COGPACK	Cognitive training game	Designed	PC	Supervised	75	2	25
Amjad et al [39]	Body and Brain Exercises	Cognitive training game	Purpose-shifted	Xbox console and Kinect	Supervised	25 to 30	5	6
Wiloth et al [40]	Physiomat	Exergame	Designed	Balance broad and screen	Supervised	90	2	10
Van Santen et al [41]	NR	Exergame	Designed	Stationary bicycle and screen	Unsupervised	NR	5	25
Karssemeijer et al [42]	NR	Exergame	Purpose-shifted	Stationary bicycle and screen	Supervised	30 to 50	3	12
Liao et al [43]	Tano and LongGood	Exergame	Designed	Kinect and VR headset	Supervised	60	3	12
Flak et al [44]	Cogmed	Cognitive training game	Designed	PC	Unsupervised	30 to 40	5	5
Hyer et al [45]	Cogmed	Cognitive training game	Designed	PC	Both	40	7	5 to 7
Park and Park [46]	CoTras	Cognitive training game	Designed	PC	Supervised	30	3	10

^a^NR: not reported.

^b^VR: virtual reality.

Half of the studies (8/16, 50%) delivered no or passive interventions to the comparison groups (eg, reading newspaper articles, surfing the internet, or watching a documentary program), whereas these groups received active interventions (eg, conventional exercises and other serious games) in 62% (10/16) of the studies (Table 3). A total of 12% (2/16) of the studies delivered both active and passive interventions as comparators. The active interventions had a duration ranging from 25 to 100 minutes. The active interventions were performed once to 7 times per week. The active comparator duration ranged from 5 to 25 weeks. The outcome of interest (ie, processing speed) was assessed using 11 different tools, with the Trail Making Test A being a frequently used tool in the included studies (7/16, 44%). The outcome of interest was measured after the intervention in all the included studies (16/16, 100%). In total, 44% (7/16) of the studies followed the participants after the interventions, and the follow-up period varied between 4 and 261 weeks. The number of participants who dropped out of the included studies ranged from 0 to 28.

**Table 3:** Characteristics of the comparators and outcomes (N=16).

Study	Comparator	Duration (minutes)	Frequency (times per week)	Period (weeks)	Outcome measures	Follow-up	Attritions, N
Finn and McDonald [31]	Control	N/A^a^	N/A	N/A	D-KEFS-NS^b^	After the intervention	7
Robert et al [32]	Control	N/A	N/A	N/A	TMT-A^c^, SCWT^d^, and WAIS-R-Dsy^e^	After the intervention; 12-week follow-up	NR^f^
Savulich et al [33]	Control	N/A	N/A	N/A	CANTAB-CRT^g^	After the intervention	0
Valdes et al [34]	Control	N/A	N/A	N/A	UFOV^h^	After the intervention; 52-, 104-, 156-, and 261-week follow-up	NR
Yang and Kwak [35]	Control	N/A	N/A	N/A	GnG^i^ and SCWT	After the intervention	0
Thapa et al [36]	Control	N/A	N/A	N/A	TMT-A and SDST^j^	After the intervention	2
Tarnanas et al [37]	Control and conventional cognitive activities	90	2	21	SCWT	After the intervention	9
Fiatarone Singh et al [38]	Control, conventional exercises+sham cognitive training, and serious games+conventional exercises	Control: 75; conventional exercises+sham cognitive training: 100; serious games+conventional exercises: 60	2	25	SDMT^k^	After the intervention; 74-week follow-up	14
Amjad et al [39]	Conventional exercises	25 to 30	5	6	TMT-A	After the intervention	6
Wiloth et al [40]	Conventional exercises	60	2	10	TMT-A	After the intervention; 12-week follow-up	26
Van Santen et al [41]	Conventional exercises	N/A	5	25	TMT-A	Midintervention and after the intervention	28
Karssemeijer et al [42]	Conventional exercises (aerobic exercises); conventional exercises (relaxation and flexibility exercises)	30 to 50	3	12	TMT-A and SCWT	Midintervention and after the intervention; 24-week follow-up	23
Liao et al [43]	Conventional exercises	60	3	12	SCWT	After the intervention	15
Flak et al [44]	Nonadaptive serious game	30 to 40	5	5	D-KEFS-CWIT1^l^ and D-KEFS-CWIT2^m^	After the intervention; 4- and 16-week follow-up	17
Hyer et al [45]	Nonadaptive serious game	40	7	5 to 7	TMT-A	After the intervention; 12-week follow-up	9
Park and Park [46]	Exergames	30	3	10	SCWT	After the intervention	0

^a^N/A: not applicable.

^b^D-KEFS-NS: Delis-Kaplan Executive Function System Test Battery-Number Sequencing.

^c^TMT-A: Trail Making Test A.

^d^SCWT: Stroop Color and Word Test.

^e^WAIS-R-Dsy: Wechsler Adult Intelligence Scale-Revised-Digit Symbol.

^f^NR: not reported.

^g^CANTAB-CRT: Cambridge Neuropsychological Test Automated Battery-Choice Reaction Time.

^h^UFOV: Useful Field of View test.

^i^GnG: go-no go.

^j^SDST: Symbol Digit Substitution Test.

^k^SDMT: Symbol Digit Modalities Test.

^l^D-KEFS-CWIT1: Delis-Kaplan Executive Function System Color-Word Interference Test 1.

^m^D-KEFS-CWIT2: Delis-Kaplan Executive Function System Color-Word Interference Test 2.

### Results of Risk of Bias Appraisal

**Figure 2:**
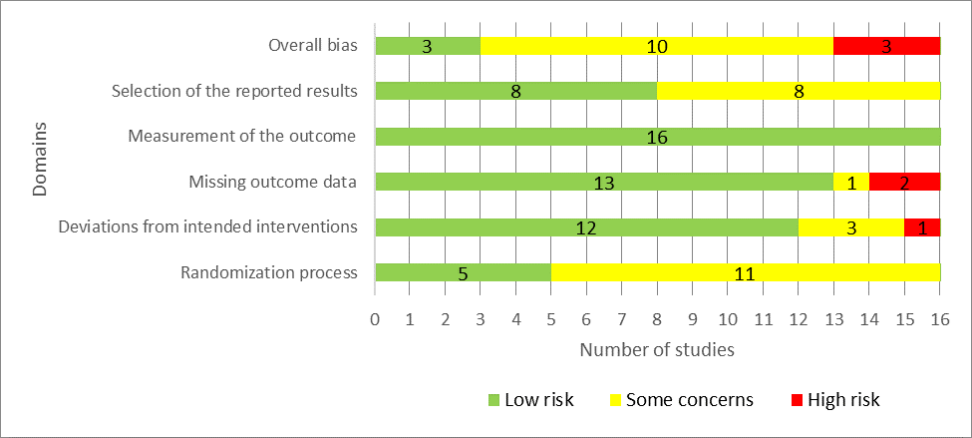
Review authors’ judgments about each “risk of bias” domain.

As shown in Figure 2, a total of 31% (5/16) of the studies were judged to have a low risk of bias in the “randomization process” domain. With regard to the “deviations from the intended interventions” domain, there was a low risk of bias in 75% (12/16) of the studies. The risk of bias because of missing outcome data was low in 81% (13/16) of the studies. All the included studies (16/16, 100%) were judged to have a low risk of bias in the “measuring the outcome” domain. In half of the included studies (8/16, 50%), the risk of bias was rated as low in the “selection of the reported results” domain. According to these judgments, only 19% (3/16) of the studies were judged to have a low risk of bias in the last domain (ie, overall bias). Reviewers’ judgments about each “risk of bias” domain for each included study are presented in Multimedia Appendix 4 [31-46].

### Results of the Studies

#### Serious Games Versus No or Passive Interventions

**Figure 3:**
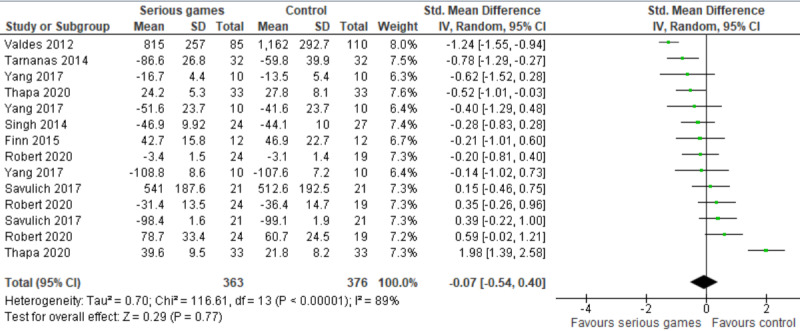
Forest plot of 8 studies (14 comparisons) comparing the effect of serious games on processing speed with that of control [31-38].

The effect of serious games was compared with that of control (no or passive interventions) in 50% (8/16) of the studies [31-38]. Passive interventions are those that do not have a known effect on the measured outcome, such as reading newspaper articles, surfing the internet, and watching a documentary program. In 50% (4/8) of these studies [32,33,35,36], more than one outcome measure was used to assess processing speed. Therefore, we included the results of all these measures in the meta-analysis to form 14 comparisons. As shown in Figure 3 [31-38], there was no statistically significant difference (*P*=.77) in processing speed between serious games and the control groups (SMD: −0.07, 95% CI −0.54 to 0.40). The statistical heterogeneity of the evidence was considerable (*P*<.001; *I*^2^=89%). The quality of the evidence was very low as it was downgraded by 6 levels owing to a high risk of bias, heterogeneity, and imprecision (Multimedia Appendix 5).

The SMDs of 2 comparisons seem to be outliers (−1.24 [34] and 1.98 [36]), although the characteristics of the studies in these comparisons were comparable with those of other studies in this meta-analysis. For this reason, we ran a sensitivity analysis to assess whether removing these outliers influenced the overall effect size and heterogeneity level. The sensitivity analysis showed that the difference in processing speed between the groups remained insignificant (*P*=.32), but the heterogeneity substantially decreased from 89% to 49%.

#### Serious Games Versus Conventional Exercises

**Figure 4:**
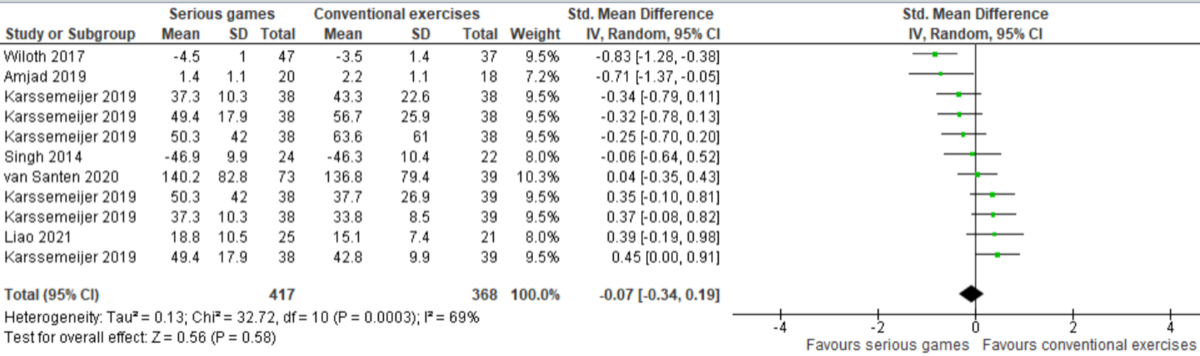
Forest plot of 6 studies (11 comparisons) comparing the effect of serious games on processing speed with that of conventional exercises [38-43].

The effect of serious games was compared with that of conventional exercises in 38% (6/16) of the studies [38-43]. Of these 6 studies, 1 (17%) compared serious games with 2 different conventional exercises (aerobic exercises and relaxation and flexibility exercises) and measured processing speed using 2 different tools [42]. Therefore, we included the results of all these comparisons and measures in the meta-analysis to form 11 comparisons (Figure 4 [38-43]). The meta-analysis showed no statistically significant difference (*P*=.58) in processing speed between the serious game and conventional exercise groups (SMD: −0.07, 95% CI −0.34 to 0.19). The statistical heterogeneity of the evidence was moderate (*P*<.001; *I*^2^=58%). The quality of the evidence was very low as it was downgraded by 6 levels owing to a high risk of bias, heterogeneity, and imprecision (Multimedia Appendix 5).

**Figure 5:**
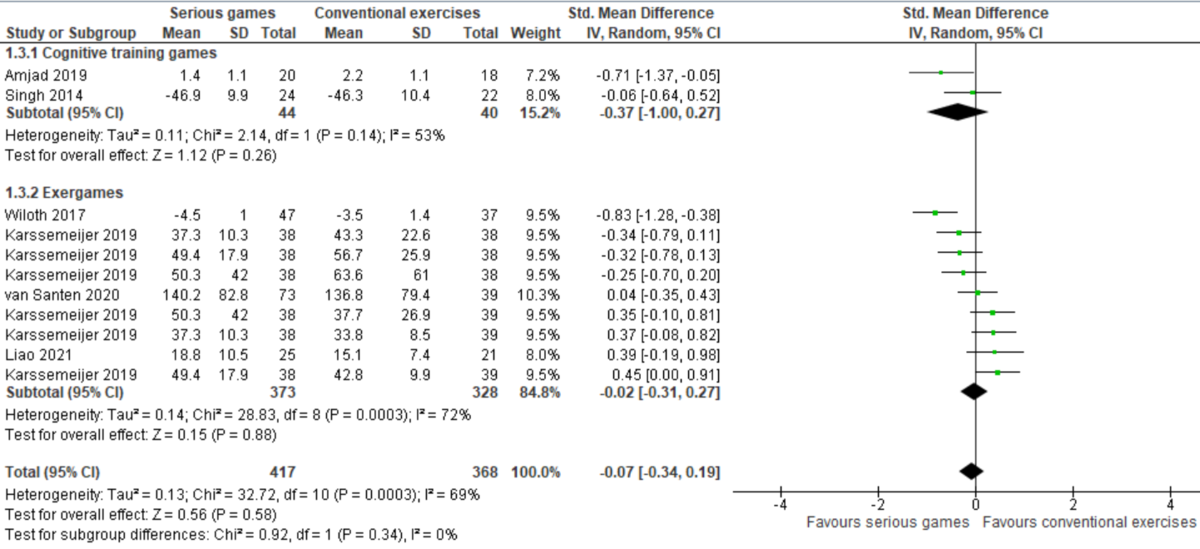
Forest plot of 6 studies (11 comparisons) comparing the effect of cognitive training games and exergames on processing speed with that of conventional exercises [38-43].

Two types of serious games were used in this comparison (ie, serious games vs conventional exercises): cognitive training games and exergames. We conducted a subgroup analysis to investigate whether cognitive training games and exergames had a different effect on processing speed (Figure 5 [38-43]). The subgroup analysis of 12% (2/16) of the studies showed no statistically significant difference (*P*=.26) in processing speed between the cognitive training game group and the conventional exercise group (SMD: −0.37, 95% CI −1.00 to 0.27). The statistical heterogeneity of the evidence was moderate (*P*=.14; *I*^2^=53%). The quality of the evidence was very low as it was downgraded by 4 levels owing to a high risk of bias, heterogeneity, and imprecision (Multimedia Appendix 5). Furthermore, the subgroup analysis of 25% (4/16) of the studies (9 comparisons) showed no statistically significant difference (*P*=.88) in processing speed between the exergame group and the conventional exercise group (SMD: −0.02, 95% CI −0.31 to 0.27). The statistical heterogeneity of the evidence was substantial (*P*<.001; *I*^2^=72%). The quality of the evidence was very low as it was downgraded by 6 levels owing to a high risk of bias, heterogeneity, and imprecision (Multimedia Appendix 5).

#### Serious Games Versus Other Serious Games

In total, 19% (3/16) of the studies assessed the effect of serious games on processing speed in comparison with that of other serious games [44-46]. The first study (1/3, 33%) compared the effect of a cognitive training game with that of exergames and found no statistically significant difference (*P*=.76) in processing speed between the groups [46]. The remaining 67% (2/3) of the studies compared the effect of cognitive training games that adjust the level of difficulty of the tasks based on an individual’s mastery in each level (ie, adaptive games) with that of the same games but without adjustment of the level of difficulty of the tasks (ie, nonadaptive games) [44,45]. Of the 2 studies, 1 (50%) showed no statistically significant difference in processing speed between the groups as measured by the Delis-Kaplan Executive Function System Color-Word Interference Test 1 (*P*=.91) and Delis-Kaplan Executive Function System Color-Word Interference Test 2 (*P*=.51) [44]. The remaining study (1/2, 50%) did not report the findings related to the outcome measure Trail Making Test A [45]. The first author of that study was contacted to obtain these findings, but he has not replied.

## Discussion

### Principal Findings

This study compiled evidence regarding the effectiveness of serious games in improving processing speed among older adults with cognitive impairment. Our review revealed that serious games are as effective as no or passive interventions and conventional exercises in improving processing speed and that there is no difference between cognitive training games and exergames when compared with conventional exercises. The nonsuperior effect of serious games over no or passive interventions and conventional exercises may have been due to the following reasons: (1) serious games in the included studies targeted cognitive abilities in general rather than processing speed specifically and (2) the sample size in most of the included studies (10/16, 62%) was small (<100).

Our findings are consistent with findings of a previous review that showed no statistically significant difference in processing speed between the cognitive training game group and the passive or active intervention group among older adults with MCI or dementia [17]. However, our findings are not consistent with findings of previous reviews [16,18-20]. Specifically, 4 reviews showed that cognitive training games [16,19,20] and exergames [18] are more effective than other interventions (passive or active) in improving processing speed among healthy older adults. This inconsistency may be attributed to the fact that (1) all these reviews focused on older adults without cognitive impairment only, whereas our review focused on older adults with cognitive impairment [16,18-20]; (2) they included quasi-experiments and pilot RCTs [18,19]; and (3) they did not compare the effect of serious games with that of a specific comparator (eg, no intervention, conventional exercises, or conventional cognitive activities) [16,18-20].

### Strengths and Limitations

#### Strengths

This study is the only one of the aforementioned reviews [16-20] that compared both serious games and their types with a specific comparator (ie, no intervention and conventional exercises) and used the GRADE approach to assess the quality of the evidence that resulted from the meta-analyses, thereby enabling the reader to draw more accurate conclusions. Given that we included only RCTs, which are the most rigorous research method for studying cause-effect relationships [47], our findings are more likely to be reliable than findings generated from reviews that included other study designs such as pilot RCTs and quasi-experiments.

The risk of publication bias in this review was minimal given that we sought to identify as many relevant studies as possible through (1) searching the most popular databases in the IT and health fields and gray literature databases, (2) conducting backward and forward reference list checking, and (3) using a well-developed search query. The risk of selection bias in this review was not a concern as the study selection, data extraction, risk of bias assessment, and quality of evidence appraisal were conducted by 2 reviewers independently.

#### Limitations

This review focused on the effectiveness of digital serious games in improving processing speed among older adults with cognitive impairment. Hence, this review cannot comment on the effectiveness (1) of nondigital serious games or those used for nontherapeutic purposes (eg, diagnosis), (2) of serious games in improving other cognitive abilities (eg, attention, learning, and memory), and (3) of serious games among other age groups or those without cognitive impairment.

The effect size estimated for each meta-analyzed study was likely overestimated or underestimated as we used postintervention data rather than the pre-post intervention change data to calculate it. We used postintervention data as most studies (12/16, 75%) did not report the mean and SD for pre-post intervention change in processing speed for each group, and the difference in processing speed between groups at baseline was not statistically significant in all studies.

This review focused only on the short-term effect of serious games by pooling only postintervention data rather than follow-up data given that the follow-up period was not consistent among the 44% (7/16) of studies that reported follow-up data. As a result, we are unable to speculate on the long-term impact of serious games on processing speed. Given that this review did not include research published before 2010, studies written in a language other than English, quasi-experiments, or pilot RCTs, it is likely that this review missed some relevant studies.

### Practical and Research Implications

#### Practical Implications

The findings of this review should be cautiously interpreted for the following reasons: (1) the quality of the evidence from all meta-analyses was very low mainly because of high risk of bias, high heterogeneity, and imprecision of the estimated total effect sizes; (2) the number of studies included in some meta-analyses was small; and (3) the sample sizes in many meta-analyzed studies were small. Consequently, until more robust evidence is available, serious games should be offered or used as a supplement rather than an alternative intervention targeting processing speed.

None of the included studies used smartphones as a platform for serious games. Smartphones are more appealing than other platforms as they are less expensive, more accessible, and more pervasive than computers and gaming consoles. In 2021, the global number of mobile devices and users was estimated to be approximately 15 billion and 7.1 billion, respectively, with these statistics likely to climb dramatically by 2025 [48]. Thus, we recommend that gaming companies develop serious games that can be played via smartphones. None of the serious games in the included studies were designed to target processing speed. Therefore, there is an urgent need to develop serious games that specifically target processing speed.

#### Research Implications

Although this review addressed the research gap related to the short-term effect of serious games on processing speed among older adults with cognitive impairment, the following research gaps need further reviews to be bridged: (1) the long-term effect of serious games, (2) the effect of serious games on other cognitive abilities (eg, attention and visuospatial skills) and other disorders (eg, attention-deficit/hyperactivity disorder, autism, and motor disabilities), and (3) the effect of serious games among people from different age groups with or without cognitive impairment.

As mentioned earlier, most studies in this review did not report the mean and SD for pre-post intervention change in processing speed. Researchers should report such information in their future publications to enable reviewers to calculate a more accurate effect size for each study. According to a previous review [18], there have been many studies conducted to assess the effect of exergames on processing speed among healthy older adults. However, only 25% (4/16) of the studies in this review investigated the effect of exergames on processing speed among older adults with cognitive impairment. Further trials are needed to address the aforementioned research gap.

In this review, only 6% (1/16) of the trials compared serious games with conventional cognitive training, and only 12% (2/16) compared adaptive serious games with nonadaptive serious games. We urge researchers to examine the aforementioned comparisons to reach more definitive conclusions. None of the included studies investigated the effect of serious games that specifically target processing speed rather than cognitive abilities in general. Future studies should use serious games that specifically target processing speed to examine their effect.

The overall risk of bias was low in only 19% (3/16) of the included trials as the remaining studies had issues mainly in the randomization process or selection of the reported results (ie, unpublished protocol or analysis plan). Future trials should improve their quality by minimizing such bias. To this end, they should be conducted and reported according to recommended guidelines or tools such as the Risk of Bias 2 [27], and they should have a large sample size that is enough to obtain the desired statistical power. As most of the included studies (14/16, 88%) were conducted in high-income countries, the generalizability of the findings of this review to lower-income countries may be restricted owing to the diversity of their cultures and socioeconomic conditions. Thus, more trials should be conducted in lower-income countries.

### Conclusions

Serious games did not have a superior effect on processing speed among older adults with cognitive impairment in comparison with no or passive interventions and conventional exercises. However, this finding should be cautiously interpreted for the following reasons: (1) the quality of the evidence from all meta-analyses was very low mainly because of high risk of bias, high heterogeneity, and imprecision of the estimated total effect sizes; (2) the number of studies included in some meta-analyses was small; and (3) the sample sizes in many meta-analyzed studies were small. Therefore, until more robust evidence is available, serious games should be offered or used as a supplement rather than as an alternative intervention targeting processing speed. Future reviews should investigate the long-term impact of serious games on other cognitive abilities and disorders among people from different age groups with or without cognitive impairment. Further trials should be undertaken to investigate the effect of serious games that specifically target processing speed rather than cognitive abilities in general.

## Appendix

Multimedia Appendix 1 PRISMA (Preferred Reporting Items for Systematic Reviews and Meta-Analyses) checklist.

Multimedia Appendix 2 Search strategy.

Multimedia Appendix 3 Data extraction form.

Multimedia Appendix 4 Reviewers’ judgments about each “risk of bias” domain for each included study.

Multimedia Appendix 5 Grading of Recommendations Assessment, Development, and Evaluation profile for comparison of serious games with control and conventional exercises regarding processing speed.

## Abbreviations

AD: Alzheimer disease
GRADE: Grading of Recommendations Assessment, Development, and Evaluation
MCI: mild cognitive impairment
PRISMA: Preferred Reporting Items for Systematic Reviews and Meta-Analyses
RCT: randomized controlled trial
SMD: standardized mean difference
