# Supplementary material for: The Effectiveness of Serious Games on Cognitive Processing Speed Among Older Adults With Cognitive Impairment: Systematic Review and Meta-analysis
Source: JMIR Serious Games. 2022 Sep 9;10(3):e36754. doi: 10.2196/36754 (PMC9508673; doi:10.2196/36754)
Supplement: Multimedia Appendix 3 [file games_v10i3e36754_app3.docx]

| **Concept** | **Definition** |
| --- | --- |
| **Study Characteristics** |  |
| Author | The first author of the study. |
| Year of publication | The year in which the study was published. |
| Country of publication | The country where the study was published. |
| Type of publication | The venue where the study was published: peer-reviewed journal articles, book chapters, dissertations, or conference proceedings |
| Type of RCT | The type of the RCT used in the study (e.g., parallel, crossover, cluster, or factorial). |
| **Population characteristics** |  |
| Number of participants | Number of people who participated in the study. |
| Number of participants in intervention group 1 | Number of participants in intervention group 1. |
| Number of participants in intervention group 2 (if any) | Number of participants in intervention group 2 if there is more than one intervention. |
| Number of participants in the control group | Number of participants in the control group. |
| Mean age | The average age of participants. |
| Sex (male) | Percentage of males in the sample. |
| Health condition of participants | What is the health condition of participants? |
| Recruitment setting | Place where participants were recruited (e.g., educational, clinical, community). |
| **Intervention characteristics** |  |
| Name of the serious game | The name given for the serious game (e.g., SPARX, Tetris, etc..). |
| Therapeutic modality | What is the therapy that the serious game provides? |
| Serious game type | What is the type of serious games?   1. Designed serious games: games that are designed with a “serious” purpose from the beginning. 2. Purpose-shifted serious games: games that were not designed as serious games but are being used for a serious purpose. 3. Modified serious games: games that are similar to purpose-shifted ones, but while purpose-shifted games are left intact, modified ones can differ from the original in terms of gameplay and characters. |
| Platform | The platform in which the serious game is implemented (e.g., mobile, tablet, PC, Console, wearable devices, etc..). |
| Duration | How long does a session of playing the serious game take, e.g., 30 mins, 120 mins, etc? |
| Frequency | How many times the serious game was used per day or week, e.g., 3 times a week, 10 times a week. |
| Period | How long the patient used the serious games (e.g., for 2 months, 6 months). |
| **Comparator** **Characteristics** |  |
| Comparator | What is the comparator (e.g., usual care, waiting list, not intervention, giving information)? |
| Duration | How long does the comparator take, e.g., 30 mins, 120 mins, etc? |
| Frequency | How many times the comparator was used per day or week, e.g., 3 times a week, 10 times a week. |
| Period | How long the patient used the comparator (e.g., for 2 months, 6 months). |
| **Outcome characteristics** |  |
| Measured outcome | What was the outcome that the study measured?  Effectiveness: Processing speed |
| Outcome measure | What is the tool used for measuring the outcome? |
| Follow-up period | When was the outcome measured? |
| Attrition | Number of the loss/dropout of participants during an experiment. |
| **Findings** |  |
| Results- Intervention- before: Mean (SD) | Results related to the outcome before delivering the intervention in the intervention group. |
| Results- control- before: Mean (SD) | Results related to the outcome before delivering the comparator in the control group. |
| Results- Intervention- after: Mean (SD) | Results related to the outcome after delivering the intervention in the intervention group. |
| Results- control- after: Mean (SD)) | Results related to the outcome after delivering the comparator in the control group. |
