# Supplementary figures and images for: The Effectiveness of Serious Games on Cognitive Processing Speed Among Older Adults With Cognitive Impairment: Systematic Review and Meta-analysis
Source: JMIR Serious Games. 2022 Sep 9;10(3):e36754. doi: 10.2196/36754 (PMC9508673; doi:10.2196/36754)

**Appendix 4 Reviewers’ judgments about each “risk of bias” domain for each included study**

**
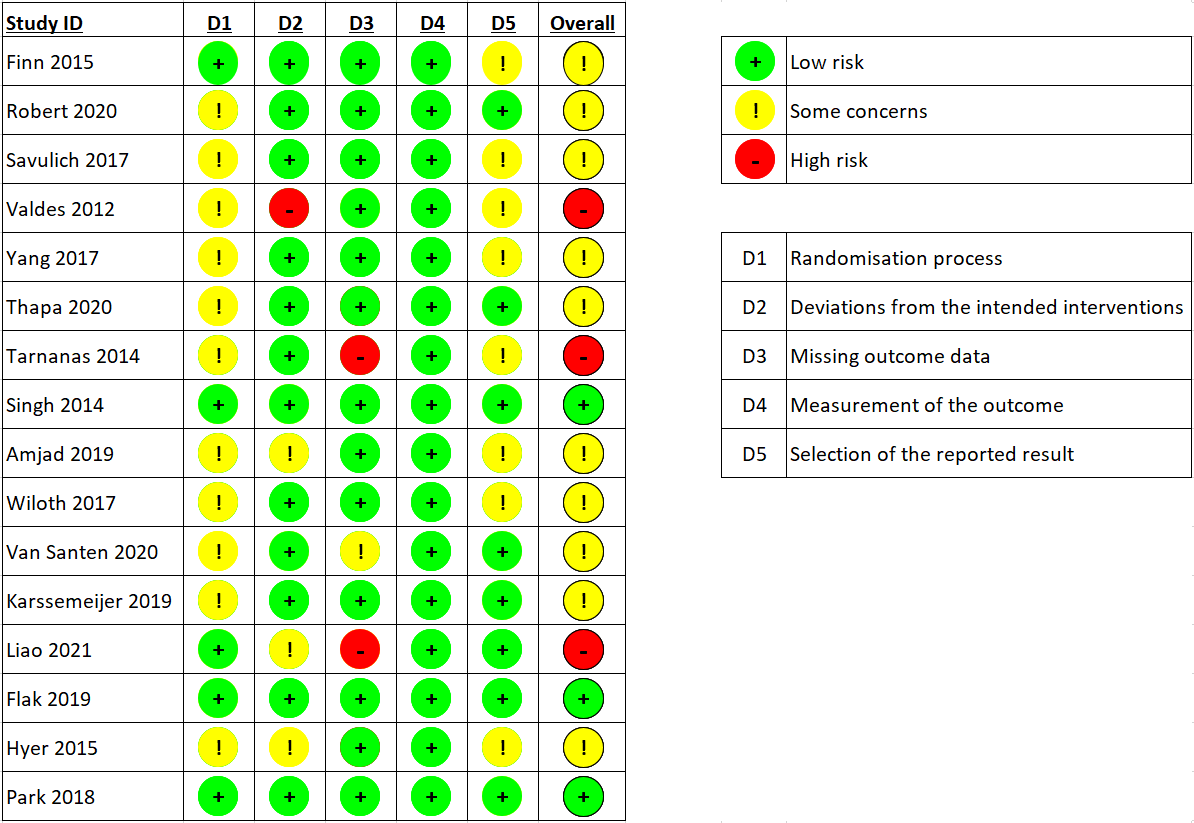
**

Supplement: Multimedia Appendix 4 [file games_v10i3e36754_app4.docx]
