# Supplementary material for: The Effectiveness of Serious Games on Cognitive Processing Speed Among Older Adults With Cognitive Impairment: Systematic Review and Meta-analysis
Source: JMIR Serious Games. 2022 Sep 9;10(3):e36754. doi: 10.2196/36754 (PMC9508673; doi:10.2196/36754)
Supplement: Multimedia Appendix 5 [file games_v10i3e36754_app5.doc]

| **Appendix 5 GRADE Profile for comparison of serious games to control and conventional exercises for processing speed** | | | | | | | | | | | |
| --- | --- | --- | --- | --- | --- | --- | --- | --- | --- | --- | --- |
| **Certainty assessment** | | | | | | | **Summary of findings** | | | | |
| **Participants (studies) Follow-up** | **Risk of bias** | **Inconsistency** | **Indirectness** | **Imprecision** | **Publication bias** | **Overall certainty of evidence** | **Study event rates (%)** | | **Relative effect (95% CI)** | **Anticipated absolute effects** | |
|  |  |  |  |  |  |  | **Control** | **Serious games** |  | **Risk with** | **Risk difference with Serious games** |
| **Serious games vs. Control** | | | | | | | | | | | |
| 739 (8 RCTs)  (14 comparisons) | very serious^a^ | very serious^b^ | not serious | very serious^c,d^ | none | ⨁◯◯◯ Very low | 376 | 363 | - | - | SMD **0.07 lower** (0.54 lower to 0.4 higher) |
| **Serious games vs. Conventional exercises** | | | | | | | | | | | |
| 785 (6 RCTs)  (11 comparisons) | very serious^e^ | very serious^f^ | not serious | very serious^c,d^ | none | ⨁◯◯◯ Very low | 368 | 417 | - | - | SMD **0.07 lower** (0.34 lower to 0.19 higher) |
| **Serious games (Cognitive training games) vs. Conventional exercises** | | | | | | | | | | | |
| 84 (2 RCTs)  (2 comparisons) | serious^g^ | serious^h^ | not serious | very serious^c,i^ | none | ⨁◯◯◯ Very low | 40 | 44 | - | - | SMD **0.37 lower** (1 lower to 0.27 higher) |
| **Serious games (Exergames) vs. Conventional exercises** | | | | | | | | | | | |
| 701 (4 RCTs)  (9 comparisons) | very serious^j^ | very serious^k^ | not serious | very serious^c,l^ | none | ⨁◯◯◯ Very low | 328 | 373 | - | - | SMD **0.02 lower** (0.31 lower to 0.27 higher) |

**CI:** confidence interval; **SMD:** standardised mean difference

#### Explanations

a. Evidence was downgraded by 2 levels because only 1 of 14 meta-analyzed studies in this comparison was judged to have a low risk of bias, this is due to issues mainly in the randomization process and selection of the reported results in the remaining studies.

b. Evidence was downgraded by 2 levels as P<0.001 and I square=89%, indicating high heterogeneity.

c. Evidence was downgraded by 2 levels because 95% CI crosses the two MID boundaries for this outcome.

d. MID for this outcome, calculated as ± 0.5 times the standardized mean difference (SMD), is ± 0.035

e. Evidence was downgraded by 2 levels because only 1 of 10 meta-analyzed studies in this comparison was judged to have a low risk of bias, this is due to issues mainly in the randomization process in the remaining studies.

f. Evidence was downgraded by 2 levels as P<0.001 and I square=69%, indicating high heterogeneity.

g. Evidence was downgraded by 1 level because there were some concerns in one of the two meta-analyzed studies due to issues mainly in the randomization process and selection of the reported results.

h. Evidence was downgraded by 1 level as P=0.14 and I square=53%, indicating moderate heterogeneity.

i. MID for this outcome, calculated as ± 0.5 times the standardized mean difference (SMD), is ± 0.185

j. Evidence was downgraded by 2 levels because none of the 9 meta-analyzed studies in this comparison was judged to have a low risk of bias due to issues mainly in the randomization process and selection of the reported results.

k. Evidence was downgraded by 2 levels as P<0.001 and I square=72%, indicating high heterogeneity.

l. MID for this outcome, calculated as ± 0.5 times the standardized mean difference (SMD), is ± 0.01.
